# Foreign

**Published:** 1841-04-10

**Authors:** 


### FOREIGN.

*Case of severe injury of the Head.* By E. J. FURNER, M. D.—Late in the evening of the 25th of last April, Mr. Lawrence was summoned by Mr. Camac, surgeon, of Seaford, to meet him in consultation upon a case of fractured skull. He arrived about twelve o'clock at night, myself and his son accompanying him, when we received the following account of the case:—The patient, William Coombs, sixteen years of age, in the employ of the Rev. James Carnegie, residing at Seaford, about six hours before our arrival had been thrown from a horse upon a hard road, and struck by the horse's feet on the left side of the head. He was taken up in a state of insensibility, and removed to his father's house, where Mr. Camac found him, perfectly insensible, with a very feeble pulse, and dilated pupils. Upon examining the head there was discovered an extensive laceration of the scalp, with fracture and depression of the temporal and parietal bones, to a very considerable extent, involving the squamous suture.

Mr. Camac, after removing two small loose pieces of fractured bone, requested that Mr. Lawrence might be sent for, the case being one of extreme danger.

Mr. Lawrence, having enlarged the wound of the scalp, was enabled, by the aid of the elevator, to remove five pieces more of depressed bone, some of which, from being driven into the brain, had forced a considerale quantity of cerebral substance, leaving a cavity, into which the finger could be passed to the depth of an inch or more: during the operation the patient cried out lustily, but became more sensible after it was completed, and soon fell into a natural sleep. The wound was carefully dressed by Mr. Camac, who has favoured me with the subsequent report of the case, and on whose skilful management the successful result must have greatly depended.

April 26th, nine o'clock. A. M.—The patient is insensible and complains of a pain in the head; reaction has taken place; pulse 100; skin hot; bowels have not been opened; eight leeches to be applied to the forehead: to take four grains of calomel directly, and an ounce of castor-oil after an hour, with a dose of mixture (containing a solution of Epsom salts) every four hours; cold lotion to be constantly applied to the head; low diet.

Five o'clock, P. M.—Bowels freely moved, with a slight improvement in all other symptoms.

27th.—Has passed a good night, and is decidedly better to-day: pain in the head partially relieved; pulse 96; skin cooler: the wound looking favourably: to have six leeches to the forehead, and continue the mixture and lotion as yesterday. The above treatment was continued with but slight alteration up to the 3d of May; the patient gradually improving.

May 3d.—The symptoms to-day have assumed a more unfavourable character, from the lad not being kept sufficiently quiet. He complains of darting pains in the head; skin hot and dry; pulse 100; the bowels open, with a healthy discharge from the wound. To be bled in the arm to six ounces; to take four grains of calomel directly; and continue the mixture, with the addition of fifteen minims of antimonal wine to each dose every four hours. The head to be shaved, and the cold lotion applied.

4th.—Passed a good night, and all the symptoms decidedly relieved; bowels freely moved; pain in the head much abated; pulse 94, and soft; skin more comfortable: a small blister to be applied to the nape of the neck, and continue the mixture every six hours.—From this time he continued to improve: the wound was daily dressed with spermaceti ointment; the hair being kept closely cut. The granulations rapidly formed, and after a few weeks dressings were discontinued for straps of adhesive plaster, and the occasional applica- of the nitrate of silver, the cold lotion being continued. The pain in the head having subsided, and the appetite returned to its natural standard, a more generous diet was allowed, with the moderate use of exercise in the open air.

November.—The wound has been for several weeks healed, but he wears, as a protection to the part, a plaster composed of equal parts of adhesive and soap plaster, and is directed to take an occasional aperient, avoiding all stimulating diet. His health and strength are perfectly restored, and his intellect not at all impaired.—*Lon. Med. Gaz.*

---

*Case of dislocation of the Tibia inwards, with the astragalus, and fracture of the Fibula.* By R. G. Coombe, M. D.—Robert Middleditch, æt. 24, on December the 5th, fell off the vat in the brewery of Messrs. Combe, Delafield, and Co., a height of sixty feet. His fall was unbroken, and he cannot describe the manner in which he alighted, as regards the position of the injured foot. Almost immediately after the accident he was conveyed to the Charing Cross Hospital, and notwithstanding the very short space of time that had elapsed from the receipt of the injury, the swelling which had supervened was very considerable. The nature of the accident was, however, easily discernible. There was a remarkable projection at the inner side of the ankle-joint, evidently caused by the dislocation of the tibia and the astragalus, with the lower fragments of the fibula attached to them; all the bones holding their relative position to each other, as was evidenced by the ability to perform flexion and extension of the foot with tolerable ease. A corresponding depression upon the opposite side of the foot existed, and about three inches above this point the fibula was fractured. The inner side of the foot was slightly inclined towards the ground, and the sole turned upwards and outwards to the same extent.

Immediately upon the patient being brought into the hospital several persevering attempts were made by Mr. Hancock to reduce the dislocation in the usual way, due attention being paid to the relaxation of the great muscles of the calf, without success. The limb was then placed in a comfortable position, a few leeches being applied, which were followed by cold lotions; and as the patient was accustomed to drink large quantities of beer, a pint of it was ordered him daily.

Nothing further was attempted up to Dec. 10th, on which day, the patient's system having been previously well relaxed by repeated doses of tartar emetic, another futile attempt was made by Mr. Hancock, assisted by Mr. Guthrie, to reduce it with the pulleys.

This attempt was only continued for a little more than a quarter of an hour, for as the extending power was being made equally from the dorsum of the foot, and from a little above the os calcis, it was thought that the cavity from which the astragalus was removed being situated between these two points, would thus be compressed, and the return of the astragalus into it be prevented. To remedy this it was determined to have a strap so constructed as, by having more purchase upon the os calcis, to allow extension to be made principally from that point. This was accordingly done, and on the following day the pulleys were again applied by Mr. Hancock and Mr. Guthrie, and gradually increased extension having been steadily maintained for upwards of an hour, the dislocation was reduced. Sloughing of the integuments over the inner ankle subsequently took place, exposing that apophysis. This case I believe to be the only one of a similar nature on record. I have in vain searched numerous surgical works, and have made inquiries of many professional men of considerable experience, with a view of ascertaing the existence of a parallel one. It seems to me to be very difficult indeed to give any explanation, in an anatomical point of view, how such an accident could occur, when we consider the great depth of the articulation between the astragalus and the scaphoid bones; the great number, strength and elasticity of the ligaments connecting the bones engaged in this dislocation; and the very beautiful and excellent provision against separation of the bones of the foot from each other, derived from the arch which these bones collectively form. It is true it may be said that the very great violence which would be applied by a fall from so great a height as sixty feet, aided perhaps by some particular position of the foot as the patient came in contact with the ground, might rupture all the ligaments connecting the tibia, the fibula, and the astragalus, to the os calcis and the scaphoid bones: but if this were the case, would not the reduction have been comparatively easy to the great difficulty that seems to have been experienced? —*Ibid.*

---

*Cases of Popliteal Aneurism.*

*Case* I.—Henry Williams, aged thirty-six, a weaver, was admitted on the 20th of May, 1830, on the recommendation of Mr. Cunningham, of Kirkcaldy, to have the femoral artery tied for popliteal aneurism. The tumour occupied the hollow of the ham—it was circumscribed in form—and, from the distinctness of its pulsation, seemed to contain little coagulum. The patient's attention had been first directed to the complaint about two months before, by an uneasy feeling of stiffness in the part, after a particularly severe day's work.

He was confined to bed, and ordered a laxative to prepare him for the operation. Next day, the pulsation had become extremely obscure, and though it slightly returned the following day, at the end of two days more it could not be perceived at all. The articular arteries were then felt much enlarged, and the tumour quickly diminished in size, while it increased in firmness, until merely a small knot the size of an olive remained. He was dismissed at his own desire on the 31st of May.

*Case* II.—William Sinclair, aged twenty-six, was admitted on the 20th of November, 1839, on account of a pulsating tumour in the popliteal space of his left leg. It was about the size of an egg, and distinctly circumscribed. The patient stated that he had first remarked the swelling and beating in the month of August, while serving as carpenter on board a whale-ship in the North Seas.

The femoral artery was tied on the 3d of December. The ligature separated on the 28th, and the patient was dismissed quite well on the 9th of January.

*Case* III.—John Lockie, aged twenty-nine, a shopkeeper in Edinburgh, was admitted on the 17th of April, 1840, on account of a large pulsating tumour occupying the ham and calf of the right leg. There was considerable œdematous swelling of the limb from the knee downwards, and over the shin-bone there were some dark-coloured spots, which had been produced by the pressure of a carefully applied flannel bandage, thus denoting a great degree of weakness in the part. The patient stated, that, about a month before admission, while walking down to Leith, he had strained the knee, and, in consequence, almost immediately afterwards perceived a beating tumour in the ham.

The artery was tied on the 30th of April, and though no unpleasant symptom followed, the swelling was slow in undergoing absorption; so that when he was dismissed, on the 3d of June, there still remained some enlargement of the limb. He nevertheless was able to resume his employment, and perform a full share of active duty; but about a fortnight ago observed a swelling in the calf of the leg, which has since opened spontaneously, and discharged a large quantity of matter, mixed with coagulated blood,—no doubt the remains of the extensive effusion which existed previously to the ligature of the vessel.

The first of these cases is curious, from the spontaneous cure occurring while the aneurism was still small and circumscribed, and the circumstances consequently unfavourable for coagulation. The second case was very similar to it, and I delayed the operation for a fortnight, to afford the chance of recovery without its performance, which might be derived from perfect rest and the pressure of a bandage. The third case seemed rather unfavourable, from the large size and sudden extension of the swelling; and the recovery was accordingly much slower than usual, though ultimately effected. It has been a question, whether an early or advanced stage of the disease is more favourable for success,—the undilated state of the anastomosing vessels being considered adverse in the former, and the quantity of extravasated blood an obstacle in the latter. From all that has fallen within my own observation, I should have no hesitation in preferring to operate at an early period, having never witnessed in my own practice the slightest unpleasant symptom of defective circulation, however small and recent the tumour might be.

Of all the operations performed for aneurism, ligature of the femoral artery is, I believe, justly regarded the easiest, either on the dead subject or on the living body, and yet the bad consequences which attend it are distinguished by their severity as well as frequency. For my own part I have been fortunate, having tied the vessel seven times for aneurism with success. But within the period of doing so, I am not aware of any case that has terminated favourably in this city, while I have either seen or heard of four that ended badly, viz. one by inflammation of the vein, one by mortification, one by hæmorrhage, and one by amputation. It is usual to attribute untoward occurrences to some peculiarity in the constitution of the part or patient; and there can be little doubt that varieties of this kind may have some influence over the result. But I feel quite sure that attention to some minute points in performing the operation has a much larger share in determining whether it shall be favourable or unfavourable.

It is established that the great sources of danger from the ligature of large arteries, are, undue laceration and separation of the connections of the vessel, whence hæmorrhage is apt to ensue; and injury to the coats of the veins, which is apt to occasion inflammation, and an obstructing coagulation, causing mortification of the limb. The subclavian artery, when tied at the external edge of the *scalenus*, lies at some distance from the vein, and neither the carotid nor the external iliac artery adheres so intimately to its accompanying venous trunk as to render it at all difficult or dangerous to pass the needle. But the femoral artery has a closer connection with the vein, and though it is felt by the operator's finger, after the fascia has been opened, round and distinct, and as if insulated from the surrounding parts, except by the loosest connections, any attempt to pass the ligature, without further dissection, either proves abortive, or if executed by force, exposes the patient to the greatest danger. I have seen a gush of dark-coloured blood proclaim transfixion of the vein; I have seen on dissection a portion of this vessel included in the ligature; and I have also seen the external coat alone grazed, as it were, by the needle, but nevertheless excited to fatal inflammation. If, on the other hand, this danger be avoided by using blunt instruments, or the finger, to detach the artery from its connections, the patient is exposed to the hardly less disastrous consequence of hæmorrhage, through ulceration or sloughing of the vessel.

To tie the femoral artery safely, the surgeon should be impressed with the conviction that the operation is one not of difficulty, but of great nicety. He should make an incision between two and a half and three inches long in the proper situation, cut through the fascia to a smaller extent, and expose the sheath of the vessels. So far he can hardly go wrong; but then, instead of hastening to pass his needle, he should, by ligature, or the temporary application of forceps, close every little vessel that discharges enough of blood to obscure distinct vision of the object he has in view. Let him now seize the sheath with dissecting forceps, and gently raising it, make a small opening by means of a straight, narrow, sharp-pointed knife. The cellular and fatty substances which envelope the vessels in variable quantity are next to be elevated and divided in successive portions, until the external coat of the artery appears quite distinct and *white*, when the needle may be passed without the slightest difficulty or danger. I am quite aware that instructions to the same effect are contained in the common books of surgery; but believing, for the reasons above cited, that sufficient attention in practice is not bestowed on the point concerned, I think it right thus seriously, and diffusively as it may seem, to repeat and enforce these directions.—*Ibid., from Prof. Syme, in the Edinburgh Monthly Journal of Medical Science.*

---

*Case of fatal Hæmorrhage in an Infant.* By Dr. Evert.—The patient was of the male sex, and eight days old; its mother had been cured of the venereal disease two years before. The child was weak when born, and its arms and legs were affected with a spotty eruption. On the 15th of April, Dr. Evert remarked a small aperture on the under lip, from which blood trickled forth. On the following day, there was very considerable hæmorrhage from the same lip, the navel, and the scrotum. On the 18th, the bleeding from the lip returned. The blood then exuded through the skin, just like the lymph which exudes when the skin is superficially abraded, and it collected in drops upon it. The child died on the 20th.—*London Med. Gaz., from Zeitschrift für die gesammte Medicin,* from a Swedish work.

---

### NEW REMEDIES.

*Paullinia.*—Dr. Gavrelle, who passed many years in the Brazils, became acquainted there with a remedy called *Paullinia,* which, when powdered and mixed with the chocolate nut, is used as a ptisan against diarrhœa and dysentery, and, generally, as a tonic. It is the fruit of a shrub of the same name (*Paullinia sorbilis,*) and of the colour of the chocolate nut; it has a peculiar smell, and a bitter taste, resembling that of rhatany. The seeds are taken out of the capsule, dried in the sun, and powdered. They contain gum, starch, a resinous matter of a brownish red colour, an oil, tannin, and a crystallizable substance with the chemical properties of caffein. Dr. Gavrelle has used the remedy in France also, with advantage, in the diarrhœa of the phthisical, chlorosis, paralysis, migraine, long convalescence, &c. The most suitable preparation is the alcoholic extract made into lozenges, syrup, pills, powders, tincture, and ointment.

*Pitch against Piles.*—Dr. Wardleworth assures us that he has used this remedy with advantage in many cases, both of external and internal piles. His usual formula consists in ordering 3½ grains of pitch to be made into pills, two of which are to be taken every evening. The well known efficacy of balsamic remedies in piles may perhaps serve to recommend these pitch-pills.—*Ibid. from Zeitschrift für die gesammte Medicin.*

